# Comparison of ordinary reverse transcription real-time polymerase chain reaction (qRT-PCR) with a newly developed one-step single-tube nested real-time RT-PCR (OSN-qRT-PCR) for sensitive detection of SARS-CoV-2 in wastewater

**DOI:** 10.1007/s11356-023-29123-2

**Published:** 2023-08-08

**Authors:** Magdaléna Rusková, Mária Bučková, Andrea Puškárová, Marianna Cíchová, Veronika Janská, Adam Achs, Zdeno Šubr, Tomáš Kuchta, Domenico Pangallo

**Affiliations:** 1 Institute of Molecular Biology, Slovak Academy of Sciences, Dúbravská Cesta 21, 845 51 Bratislava, Slovakia; 2 Water Research Institute, Nábrežie Arm. Gen. L. Svobodu 5, 812 49 Bratislava, Slovakia; 3 Biomedical Research Center, Slovak Academy of Sciences, Institute of Virology, Dúbravská Cesta 9, 845 05 Bratislava, Slovakia; 4 Department of Microbiology, Molecular Biology and Biotechnology, Food Research Institute, National Agricultural and Food Centre, Priemyselná 4, 824 75 Bratislava, Slovakia; 5 Caravella, s.r.o., Tupolevova 2, 851 01 Bratislava, Slovakia

**Keywords:** Wastewater, One-step single-tube nested reverse transcription real-time PCR, Encapsidated RNA mimic system, Polyethylene glycol, SARS-CoV-2, Epidemic surveillance

## Abstract

Wastewater monitoring has proven to be an important approach to detecting and controlling the development of the SARS-CoV-2 pandemic. Various tests based on reverse transcription real-time PCR (qRT-PCR) have been developed and used for the detection of SARS-CoV-2 in wastewater samples. In this study, we attempted to increase the sensitivity of qRT-PCR by developing a one-step single-tube nested qRT-PCR assay (OSN-qRT-PCR). Two variants were developed, oriented to nucleocapsid phosphoprotein gene (*N*) and to spike protein gene (*S*), respectively. The performance of conventional qRT-PCR assays oriented to these genes with two novel OSN-qRT-PCR assays were firstly optimized using wastewater artificially contaminated with two encapsidated RNA mimic systems harboring a portion either *N* or *S* gene (ENRM and ESRM, respectively). The assays were coupled to a polyethylene glycol–based RNA precipitation/extraction method and applied to detect SARS-CoV-2 in wastewater samples from four cities in Slovakia. Both novel OSN-qRT-PCR assays demonstrated higher detection rates than the ordinary qRT-PCR counterparts. The virus levels in the analyzed wastewater samples had a high or very high relation with the numbers of clinical cases in the monitored regions. In fact, correlation with a 3-, 4-, or 5-day temporal offset was revealed. The OSN-qRT-PCR assays demonstrated robustness, mainly in samples with low viral loads.

## Introduction

Severe acute respiratory syndrome coronavirus 2 (SARS-CoV-2), which emerged in Wuhan, China, in 2019, is the etiological agent of the COVID-19 (coronavirus disease 2019) pandemic that affected humans and animals worldwide (El-Sayed and Kamel 2021; Kamel et al. 2023). The most common accepted human-to-human transmission route of this virus was through coughing, sneezing, and the spread of respiratory droplets or aerosols (Lotfi et al. 2020; El-Sayed et al. 2021).

The COVID-19 pandemic showed that effective, sensitive, and robust surveillance tools are necessary to detect the pathogenic agent in anthropogenic environments. The timely detection of SARS-CoV-2 can be an important tool in the hands of operators in order to implement targeted actions for the safeguard of public health. Various systems, based on different principles, were applied to monitor the presence of SARS-CoV-2. For example, a smart health monitoring system was developed using the Internet of Things (IoT) technology which is capable of monitoring blood pressure, heart rate, oxygen level, and temperature of a person. This system is helpful for rural areas or villages where nearby clinics can be in touch with city hospitals about their patient health conditions (Bhardwaj et al. 2022). Nucleic acid detection by isothermal amplification and the collateral cleavage of reporter molecules by CRISPR-associated enzymes is another promising alternative to quantitative PCR. The specific high-sensitivity enzymatic reporter unlocking (SHERLOCK) assay using the enzyme CRISPR-Cas from *Leptotrichia wadei* provides a promising method for sensitive and specific detection of SARS-CoV-2 viral RNA. Assay based on SHERLOCK system is valid for clinical test and should facilitate SARS-CoV-2 detection in settings with limited resources (Patchsung et al. 2020).

On the other side, a monitoring system of wastewater based on real-time PCR can be considered also as a valuable approach to reveal the onset of a pathogen, to control its presence, and to follow the epidemic trend (Hamouda et al. 2021; Pandey et al. 2021).

The first step at the detection of the virus in wastewater is the concentration of virus particles, followed by the RNA extraction. Ultracentrifugation, precipitation, flocculation, and filtration are the most used techniques to concentrate SARS-CoV-2 from wastewater samples (Rusiñol et al. 2020; Mazumder et al. 2022). However, their performance is apparently affected by the experimental conditions, as different researchers, using surrogate viruses or plasmids, found different efficiency of the individual methods (Barril et al. 2021; Bertrand et al. 2021; Colosi et al. 2021). Finally, polyethylene glycol (PEG) precipitation became widely utilized in many laboratories because of its ease of use and because it does not require specific equipment such as an ultracentrifuge (Alexander et al. 2020).

Several reverse transcription real-time PCR assays (qRT-PCR) were developed for detecting SARS-CoV-2 from wastewater samples. Many of these assays were oriented to gene sequences of nucleocapsid phosphoprotein (*N*) of the virus and only few to other genes including spike protein gene (*S*) (Bivins et al. 2021; Saawarn and Hait 2021). An improvement in sensitivity of qRT-PCR was achieved by developing its nested variant, but this approach was applied mainly for clinical samples (Wang et al. 2020; Yip et al. 2020). Nested PCR assays are composed of two sets of primers (external and internal), and the product of PCR with external primers acts as a template for PCR with internal primers. In order to avoid opening the tubes after the first PCR and pipetting the product into new tubes, which is not only laborious but also risky in terms of contamination of the laboratory environment, a technique was developed in which both reactions run in the same tube (single-tube nested real-time PCR). Here, the external primers work at a higher annealing temperature than the internal ones, and switching between the two PCR steps is achieved by a change in the annealing temperature (Minarovičová et al. 2011; Costa et al. 2012). The process has been also adapted to detect RNA as qRT-PCR (Wang et al. 2020; Yip et al. 2020). However, the nested PCR assays applied to wastewater samples until now used the conventional procedure with gel electrophoresis (Rosa et al. 2020; Haramoto et al. 2020).

Another important aspect, regarding the development of a PCR assay, is the use of a reliable positive control (standard) in order to optimize the conditions of nucleic acid extraction and consequent amplification. Various commercial standards are available in the market, and plasmids harboring SARS-CoV-2 sequences can be created (Petrovan et al. 2020; Toptan et al. 2020; Chik et al. 2021). However, a valuable option is the construction of an encapsidated mimic system containing the desired SARS-CoV-2 sequences (Peyret et al. 2022). These encapsidated mimics are similar to the target viral particles and with their use facilitates simulation of a real situation. This kind of mimic systems can be produced in large amounts for satisfying the experimentation and optimization needs.

In this study, we present two variants of a one-step single-tube nested reverse transcription real-time PCR assay (OSN-qRT-PCR) designed on already known and used SARS-CoV-2 qRT-PCR assays oriented to nucleocapsid phosphoprotein gene (*N*) and spike protein gene (*S*) (Lu et al. 2020; https://covid19.edgebioinformatics.org/#/assayValidation). The sensitivity of the two OSN-qRT-PCR assays together with two ordinary qRT-PCR assays oriented to the same targets was determined with the aid of dedicated encapsidated RNA mimic systems containing a fragment of either *N* or *S* gene (ENRM and ESRM, respectively). Then, the four PCR assays, in combination with a PEG-based concentration and a chaotropic RNA extraction method, were compared at detection of SARS-CoV-2 in wastewater samples collected from four different wastewater treatment plants in Slovakia.

## Materials and methods

### Primer design

Several sets of primers and probes were designed using the software Primer3Plus (https://dev.primer3plus.com/index.html). The first group of primers and probes was oriented to the SARS-CoV-2 spike protein gene (*S*) (Rusková et al. 2022). The second group of primers and probes was oriented to nucleocapsid phosphoprotein gene (*N*; accession number: MZ054877). The primers and probe sequences, together with characteristics of corresponding PCR assays, are described in Table 1.

**Table 1:** Oligonucleotide primers and probes for ESRM/ENRM construction and SARS-CoV-2 detection used in this study

Aim	Primer/probe name	Sequence (5′–3′)	Amplicon size	Reference
ESRM and ENRM construction				
Amplification of SARS-CoV-2 *S* gene fragment	IF-CoS For	*ATC AGG CCG GCC GGG GTA CCA TTG GCA AAA TTC AAG ACT CAC	447 bp	Rusková et al. (2022)
	IF-CoS Rev	*GTG CAC AAC AAC GTT GGT ACC AGG AGC AGT TGT GAA GTT C		
Amplification of SARS-CoV-2 *N* gene fragment	IF-CoN For	ATC AGG CCG GCC GGG GTA CCA TGT CTG ATA ATG GAC CCC	573 bp	This work
	IF-CoN1 Rev	GTG CAC AAC AAC GTT GGT ACC ACT GTT GCG ACT ACG TGA TG		
Recombinant analysis	NCuniFor	GAG GCA ATT TGT GCT TCA ATG G	779 bp plus insert	Šubr et al. (2010)
	NCuniRev	CGC TTA ACT CCT TCA TAC CAA G		
PCR detection assays				
qRT-PCR (S)	HOT_Spike_Fw	AGT GCA AAT TGA TAG GTT GATC	88 bp	Rusková et al. (2022)
	HOT_Spike_Rv	TCT GAT TTC TGC AGC TCT AAT TA		
OSN-qRT-PCR (S)	LANL_May4.1_Fw	CRC GTC TTG ACA ARG TTG AGG CT	155 bp	Pandey et al. (2021)
	LANL_May4.1_Rv	TAC ACA CTC TGA CAT TTT AST AGC AGC		
OSN-qRT-PCR (S)	Inner_Spike_Fw	AGT GCA AAT TGA TAG GTT G	85 bp	Rusková et al. (2022)
	Inner_Spike_Rv	GAT TTC TGC AGC TCT AAT TA		
qRT-PCR (S) and OSN-qRT-PCR (S)	P-LANL_4.1	FAM-GGC AGA CTT CAA AGT TTG CA-BHQ1	Probe	Rusková et al. (2022)
qRT-PCR (N)	N3-F	GGG AGC CTT GAA TAC ACC AAA A	72 bp	Lu et al. (2020)
	N3-R	TGT AGC ACG ATT GCA GCA TTG		
OSN-qRT-PCR (N)	N3_nested_Fw	CGG CAT CAT ATG GGT TGC AAC TGA	150 bp	This work
	N3_nested_Rv	CCT CTG CTC CCT TCT GCG TAG A		
OSN-qRT-PCR (N)	Inner_N3_Fw	AGC CTT GAA TAC ACC AAA A	67 bp	This work
	Inner_N3_Rv	TAG CAC GAT TGC AGC ATT		
qRT-PCR (N) and OSN-qRT-PCR (N)	N3-P	FAM-AYC ACA TTG GCA CCC GCA ATC CTG-BHQ1	probe	Lu et al. (2020)

^*^Sequence of the vector adjacent to the cloning site (for hybridization at in-fusion cloning) is underlined

### Construction of the encapsidated S and N RNA mimics (ESRM and ENRM)

Fragments of the SARS-CoV-2 *S* and *N* genes (447 nt and 573 nt, respectively) were amplified from cDNA of the isolate hCoV-19/Slovakia/SK-BMC5/2020 (GISAID.org accession ID EPI_ISL_417879). PCR was performed using EX Taq DNA polymerase (Takara, Shiga, Japan) and primers specified in Table 1 under the following conditions: initial denaturation at 94 °C for 3 min followed by 40 cycles of denaturation at 94 °C for 15 s, annealing at 56 °C for 20 s, elongation at 72 °C for 30 s, and a final extension at 72 °C for 3 min (Rusková et al. 2022).

A plum pox virus (PPV) genome–based vector pAD-agro for transient expression in plants (Achs et al. 2022) was used to construct the encapsidated S and N RNA mimics (ESRM and ENRM). Particular amplimers were purified from agarose gel by Wizard SV gel and PCR Clean-up System (Promega, Madison, Wisconsin, USA) and inserted into *Kpn*I-digested pAD-agro using In-Fusion HD Cloning Kit (Takara). After transformation of *E. coli* JM109, the plasmid DNA was isolated by QIAprep Spin Miniprep Kit (Qiagen, Hilden, Germany), verified by sequencing and electroporated into *Agrobacterium tumefaciens* EHA105. Overnight cultures of agrobacteria were sedimented by centrifugation (16,000* g* for 1 min) and resuspended in 10 mM MES pH 5.6, 10 mM MgCl_2_, 200 μM acetosyringone to reach final OD_600_ of ~ 0.1. The suspension was incubated for 2 h at 20 °C and infiltrated into two leaves of 3–4-week-old *Nicotiana benthamiana* plants by a needleless syringe (approximately 100 μl per plant). The plants were cultivated in an insect-free room under controlled conditions (temperature 20–22 °C, 12 h light/dark photoperiod). The presence of PPV in symptomatic plant tissues was confirmed by Western blotting using a specific polyclonal antibody (Šubr and Matisová 1999). Further analysis by RT-PCR was performed after total leaf RNA isolation (by NucleoSpin RNA Plant Kit; Macherey–Nagel) using AMV reverse transcriptase (Promega) and PCR with primers NCuniFor/NCuniRev (Šubr et al. 2010) spanning the cloning site of pAD-agro (Table 1). Amplification products were sequenced to verify the inserted fragments.

ESRM/ENRM were purified 2 weeks after infiltration by the modified protocol of Laín et al. 1988. Virions were extracted from plant tissues with two volumes (2 ml per gram of tissue) of extraction buffer (18 mM McIlvain citrate–phosphate buffer pH 7.0 with 0.2% thioglycolic acid, 10 mM DIECA, 0.5 M urea, 2 mM EDTA, 1 mM PMSF) and 1/3 volume of chloroform. Phase separation was achieved by low-speed centrifugation at 1520* g* for 30 min and then, the water phase was ultracentrifuged at 57,000* g* for 2 h. The sediment was resuspended in 100 mM sodium borate buffer pH 8.2 with 10 mM EDTA and clarified by low-speed centrifugation (1 520* g* for 15 min). Sucrose was added to the solution to the final concentration of 20% and another round of ultracentrifugation (57,000* g* for 2 h) was performed. The purified ESRM/ENRM were obtained by resuspension of the sediment in a small volume of 10 mM Tris–HCl pH 8 with 1 mM EDTA and centrifugal clarification (16,000* g* for 5 min). The products were stored in aliquots at − 20 °C until used, but for a maximum of 3 months.

### Ordinary reverse transcription real-time PCR

Ordinary reverse transcription real-time PCR (qRT-PCR) assays were performed in 20 μl of total reaction volume. Each reaction tube comprised 5 μl of RNA solution, 10 μl of Luna universal probe one-step RT-qPCR mixture (New England Biolabs, Beverly, Massachusetts, USA), 1 μl of Luna warm start RT enzyme Mix (New England Biolabs), 400 nmol/l of primers HOT_Spike_Fw/HOT_Spike_Rv, and 200 nmol/l of probe P_LANL_4.1 (Table 1). The same procedure was used for the qRT-PCR assay oriented to *N* gene, but the oligonucleotides were N3-F/N3-R (primers) and N3-P (probe; Table 1). The real-time PCR cycler used was Applied Biosystems QuantStudio 1 (Thermo Fisher, Waltham, Massachusetts, USA), and the temperature program consisted of the following steps: 55 °C for 10 min, 95 °C for 1 min, 45 cycles of 95 °C for 10 s, and 60 °C for 1 min. The fluorescence signal in FAM channel was collected at the end of each cycle. Each run contained positive (ESRM or ENRM) and negative controls. Data were collected and analyzed using QuantStudio Design and Analysis Software v1.5.2 (Thermo Fisher). Cycle threshold (*C*_*t*_) values were calculated at the automatic threshold setting. The fluorescence signal in positive samples showed a typical S-shaped amplification curve, and a sample was considered positive when *C*_*t*_ ≤ 38.

### One-step single-tube nested reverse transcription real-time PCR

One-step single-tube nested qRT-PCR (OSN-qRT-PCR) was carried out in mixtures of the same chemical composition as ordinary qRT-PCR but with different primers. The reaction mixture of OSN-qRT-PCR oriented to *S* gene comprised the outer primers LANL_MAY_4.1_Fw/LANL_MAY_4.1_Rv and the inner primers Inner_Spike_Fw/Inner_Spike_Rv (Table 1). In case of OSN-qRT-PCR oriented to *N* gene, the reaction mixture comprised the outer primers N3_nested_Fw/N3_nested_Rv and the inner primers Inner_N3_Fw/Inner_N3_Rv (Table 1). For both OSN-qRT-PCR assays, the same amplification program was used, which included the following steps: 55 °C for 10 min, 95 °C for 1 min, 10 cycles of 95 °C for 15 s, 64 °C for 30 s, and 72 °C for 40 s, followed by 40 cycles of 95 °C for 15 s and 55 °C for 30 s. The fluorescence signal in FAM channel was collected at the end of each cycle. A sample was considered positive for *C*_*t*_ ≤ 30 in the second phase of amplification.

### Assessing the sensitivity of PCR assays

RNA isolated from ENRM and ESRM by NucleoSpin RNA Virus Kit (Macherey–Nagel, Düren, Germany) was used as a standard for the sensitivity of the four PCR assays (N qRT-PCR, S qRT-PCR, N OSN-qRT-PCR, and S OSN-qRT-PCR). A standard curve was generated using tenfold serial dilutions (10^−1^ to 10^−9^) of ENRM or ESRM RNA, and RNase-free water was used as a negative control. The starting concentrations of ENRM and ESRM were 5.14 ng/μl and 3.41 ng/μl, respectively. All the amplification reactions were run in triplicates in two independent assays.

### Wastewater samples

Samples of influent wastewater from four wastewater treatments plants (WWTP) were analyzed. WWTP in four medium-sized cities in Slovakia were included in the study. Bardejov is a city with approximately 30,000 inhabitants located in North-Eastern Slovakia, well known for nearby spa Bardejovské Kúpele. Poprad is a city with approximately 50,000 inhabitants located in North-Eastern Slovakia located near a popular High Tatra Mountain Park. Kysucké Nové Mesto, an industrial city with 15,000 inhabitants located in North-West Slovakia, was chosen due to its high incidence and morbidity of COVID-19. Komárno is a city with 30 000 inhabitants in South Slovakia, aside of tourism near the border with Hungary. Influent wastewater 24 h composite samples were collected from four WWTPs located in these four cities (Bardejov, Kysucké Nové Mesto, Komárno, Poprad). The composite samples were taken at the inlet of wastewater treatment plants, after the screening and grit removal steps and at a site that was well mixed. Twenty-four hour flow/time-dependent composite sample of 1000 ml was refrigerated at 4 °C during the sampling period. The samples in each locality were recovered four times during the month of October 2021 (on 6, 13, 20, and 27 October 2021). Technical data on WWTP are provided in Table 2.

**Table 2:** Wastewater treatment plant characteristics

Wastewater treatment plant	Population served	Inlet volume* of 24 h (m^3^)	T (°C)*
Bardejov	25,700	5100–6180	12.9–14.0
Kysucké Nové Mesto	17,300	2110–2320	14.5–17.9
Komárno	25,400	10,200–10,500	16.0–18.0
Poprad	89,000	32,000–37,000	11.5–12.5

^*^Parameters measured during sampling during October 2021

Collected wastewater samples were transferred on ice to the laboratory within a few hours, kept refrigerated at 4 °C and processed within 24 h.

### Sample processing

A protocol of Wu et al. 2020 with some modifications was used. Portions of 50 ml of wastewater samples were clarified from particulate biomass by centrifugation for 30 min at 4 500* g* and 4 °C without braking (Rusková et al. 2022). Virus particles were precipitated by 10% PEG 8000 in presence of 400 mM NaCl for 15 min at 20 °C and permanent agitation. Subsequently, the precipitate was sedimented by centrifugation for 45 min at 12,000* g* and 4 °C without braking. The supernatant was carefully removed, and the sediment was dissolved in 800 µl of TRIzol reagent (Molecular Research Center, Cincinnati, Ohio, USA) by vortexing for 15 s. Then, it was centrifuged at 2000* g* for 10 s; the supernatant was transferred to a clean 1.5-ml tube and used for RNA extraction. RNA was isolated using NucleoSpin RNA Virus Kit (Macherey–Nagel) according to the manufacturer’s instructions. The extracted RNA solution was used immediately for the detection of SARS-CoV-2 using diverse qRT-PCR assays or stored at − 70 °C for a maximum of 4 weeks.

### Data on clinical cases

Data on clinical cases from September 22 to November 10, 2021, from Bardejov, Poprad, Kysucké Nové Mesto, and Komárno counties was downloaded from https://github.com/ (Institute for Healthcare Analyses of the Ministry of Health of the Slovak Republic in 2022).

### Statistical analysis

The data are given as mean of 3 to 5 experiments ± standard deviation (SD). The differences between the given groups were tested for statistical significance using Student’s *t*-test (*p < 0.05; **p < 0.01; ***p < 0.001). Viral load in wastewater determined by qRT-PCR and OSN-qRT-PCR assays were compared using a Pearson’s correlation test (Acosta et al. 2021). Pearson’s correlation was calculated also between wastewater data and clinical data (Wu et al. 2022).

## Results

### Use of encapsidated RNA mimics to evaluate the PCR assays

The two encapsidated RNA mimics (ESRM and ENRM) were used to optimize the PCR assays and to generate the corresponding standard curves. ESRM and ENRM contain fragments of the *S* and the *N* gene of SARS-CoV-2, respectively. These mimic constructs can be useful also as robust positive controls that attest the successful performance of PCR. In fact, diverse SARS-CoV-2 gene fragments can be inserted in the PPV genome if necessary for future PCR assay development.

**Fig. 1:**
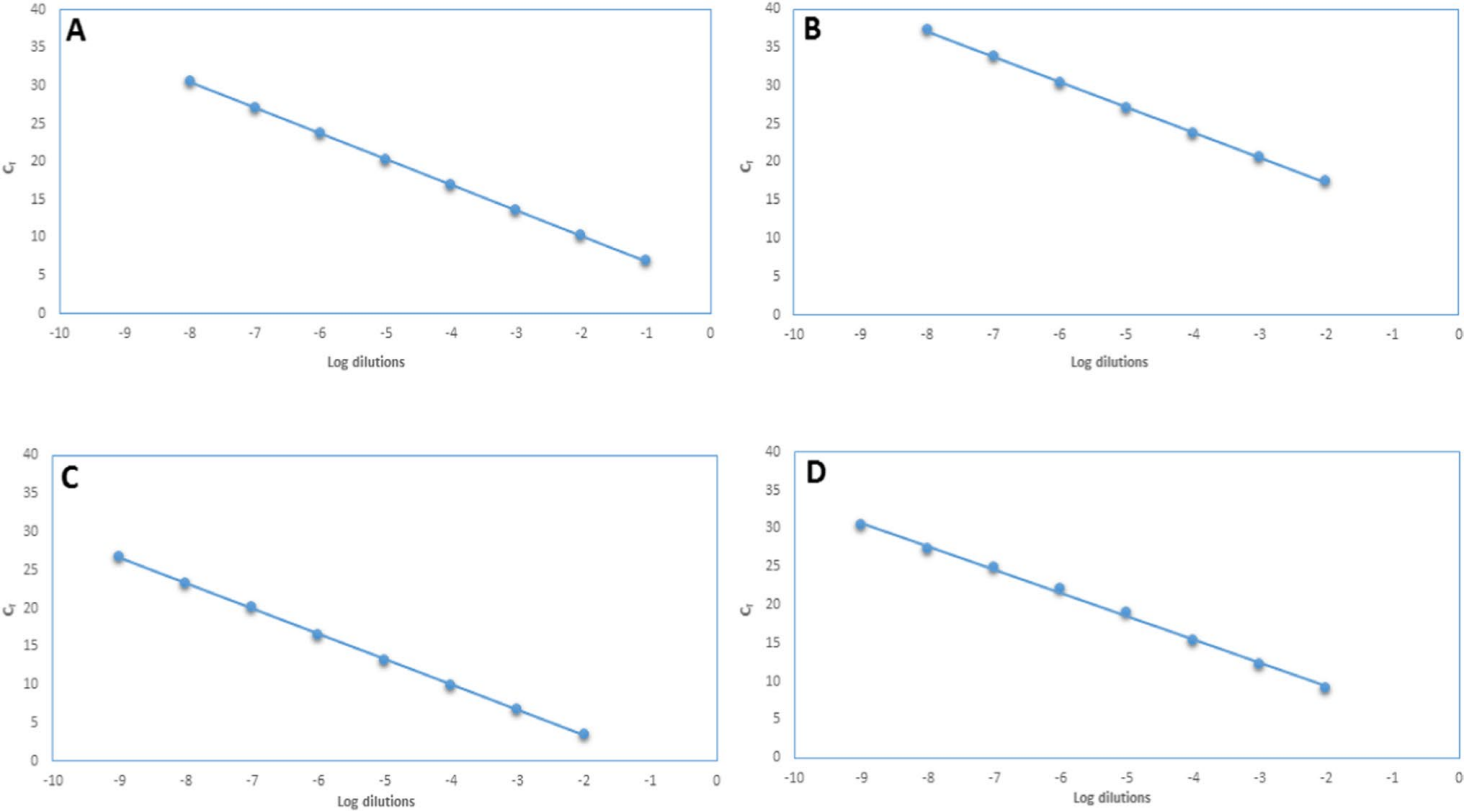
The standard curves of (**A**) N gene qRT-PCR, (**B**) S gene qRT-PCR, (**C**) N gene OSN-qRT-PCR, and (**D**) S gene OSN-qRT-PCR, plotted with *C*_*t*_ values from 514 to 5.14 × 10^−8^ ng/µL for *N* and from 341 to 3.41 × 10.^−8^ ng/µL for *S* of ENRM (**A**, **C**) and ESRM (**B**, **D**)

With serially diluted RNA mimics, real-time PCR assays were run, and based on *C*_*t*_ values, linear calibration curves were plotted (Fig. 1A and C). The regression analysis for ENRM quantification showed *R*^2^ of 1 and 0.996, with efficiency of 98.59% and 100.47% for N qRT-PCR and N OSN-qRT-PCR, respectively. Similar performance was recorded for ESRM quantification (Fig. 1B and D), for which *R*^2^ values of 1 and 0.999 were calculated, with efficiency of 101.32% and 103.29% for S qRT-PCR and S OSN-qRT-PCR, respectively. The slopes of the standard curves for quantification of N gene and S gene were − 3.356 for N qRT-PCR, − 3.311 for N OSN-qRT-PCR, − 3.291 for S qRT-PCR, and − 3.246 for S OSN-qRT-PCR. The assay limit of quantification (ALOQ, the lowest copy number detected in 100% of assays) for the two OSN-qRT-PCR assays was 1 copy/reaction and for the two qRT-PCR was tenfold higher (10 copies/reaction). OSN-qRT-PCR method was found to be highly sensitive, which is a good prerequisite for improved detection of SARS-CoV-2.

### Comparison of qRT-PCR and OSN-RT-qPCR assays for detection of SARS-CoV-2

The newly developed OSN-RT-qPCR assays were compared to corresponding ordinary qRT-PCR by analyzing wastewater samples. By analyzing 4 wastewater samples from each of 4 cities in Slovakia, no positive results were obtained by S qRT-PCR, but all of 16 analyzed wastewater samples were found to be positive by S OSN-qRT-PCR. All 16 analyzed wastewater samples were also found to be positive by both N qRT-PCR and N OSN-qRT-PCR assays. Table 3 presents the cycle threshold (*C*_*t*_) values and numbers of gene copies (GC) of the detected genes in four WWTP at given dates, as read from corresponding calibration curves. Concentration of nucleocapsid phosphoprotein gene (*N*) and spike protein gene (*S*) of SARS-CoV-2 in wastewater samples ranged from ND to 3.37 × 10^8^ GC/ml (for *S*) and 7.6 × 10^4^ to 5.0 × 10^6^ GC/ml (for *N*). Primers for *S* detected by conventional qRT-PCR (HOT_Spike_Fw/HOT_Spike_Rv) were not suitable for the environmental wastewater samples. The highest positivity was detected in the sample from Bardejov (27 of October); by conventional qRT-PCR, we detected 1.54 × 10^6^ GC/ml (for N), and by OSN-qRT-PCR, we detected 5 × 10^6^ GC/ml (for N) and 3.37 × 10^8^ GC/ml (for S). Nevertheless, that conventional qRT-PCR is the gold standard for laboratory testing viruses, OSN-qRT-PCR (especially for N gene) can detect SARS-CoV-2 and has the higher sensitivity and specificity, and it is the more suitable assay for the detection of samples with low viral load. Pearson’s correlation was calculated among N qRT-PCR, N OSN-qRT-PCR, and S OSN-qRT-PCR, and all correlations were positive. Highest correlations were seen when comparing viral load detected by N OSN-qRT-PCR and S OSN-qRT-PCR (*r* = 0.96). Very high correlation (0.8 ≤ *r* ≤ 1.0) was between N qRT-PCR and N OSN-qRT-PCR (*r* = 0.93) and also between N qRT-PCR and S OSN-qRT-PCR (*r* = 0.92).

**Table 3:** The cycle threshold (*C*_*t*_) values and number of gene copies (GC) ± standard deviation (*n* = 3)

Sample	N gene				S gene			
	qRT-PCR		OSN-qRT-PCR		qRT-PCR		OSN-qRT-PCR	
	*C*_*t*_	*Q* (GC/ml)	*C*_*t*_	*Q* (GC/ml)	*C*_*t*_	*Q* (GC/ml)	*C*_*t*_	*Q* (GC/ml)
10.6.2021 Bardejov	33.05 ± 0.015	1.23 × 10^6^	25.22 ± 0.004	1.92 × 10^6^	*ND	ND	23.1 ± 0.003	1.17 × 10^8^
13.10.2021 Bardejov	35.11 ± 0.016	3.02 × 10^5^	27.625 ± 0.003	3.76 × 10^5^	ND	ND	25.32 ± 0.004	2.19 × 10^7^
20.10.2021 Bardejov	32.74 ± 0.014	1.53 × 10^6^	24.44 ± 0.005	3.26 × 10^6^	ND	ND	21.89 ± 0.005	2.92 × 10^8^
27.10.2021 Bardejov	32.73 ± 0.013	1.54 × 10^6^	23.81 ± 0.002	5.00 × 10^6^	ND	ND	21.7 ± 0.002	3.37 × 10^8^
6.10.2021 Kysucké Nové Mesto	34.85 ± 0.015	3.61 × 10^5^	25.46 ± 0.002	1.63 × 10^6^	ND	ND	23.27 ± 0.004	1.03 × 10^8^
13.10.2021 Kysucké Nové Mesto	35.33 ± 0.014	2.60 × 10^5^	27.24 ± 0.004	4.88 × 10^5^	ND	ND	23.11 ± 0.002	1.16 × 10^8^
20.10.2021 Kysucké Nové Mesto	35.41 ± 0.013	2.46 × 10^5^	27.56 ± 0.003	3.93 × 10^5^	ND	ND	25.22 ± 0.006	2.37 × 10^7^
27.10.2021 Kysucké Nové Mesto	35.73 ± 0.012	1.98 × 10^5^	28.1 ± 0.003	2.72 × 10^5^	ND	ND	26.19 ± 0.005	1.14 × 10^7^
6.10.2021 Komárno	37.13 ± 0.014	7.60 × 10^4^	29.07 ± 0.005	1.41 × 10^5^	ND	ND	25.7 ± 0.005	1.65 × 10^7^
13.10.2021 Komárno	37.01 ± 0.013	8.25 × 10^4^	29.25 ± 0.004	1.25 × 10^5^	ND	ND	27.01 ± 0.004	6.14 × 10^6^
20.10.2021 Komárno	35.06 ± 0.015	3.13 × 10^5^	27.53 ± 0.003	4.01 × 10^5^	ND	ND	23.88 ± 0.003	6.50 × 10^7^
27.10.2021 Komárno	35.07 ± 0.014	3.11 × 10^5^	27.21 ± 0.006	4.98 × 10^5^	ND	ND	24.76 ± 0.003	3.35 × 10^7^
6.10.2021 Poprad	35.06 ± 0.012	3.13 × 10^5^	27.44 ± 0.005	4.26 × 10^5^	ND	ND	24.51 ± 0.006	4.04 × 10^7^
13.10.2021 Poprad	36.74 ± 0.015	9.93 × 10^4^	28.4 ± 0.004	2.22 × 10^5^	ND	ND	25.9 ± 0.005	1.42 × 10^7^
20.10.2021 Poprad	35.99 ± 0.012	1.66 × 10^5^	27.9 ± 0.003	3.12 × 10^5^	ND	ND	25.76 ± 0.004	1.58 × 10^7^
27.10.2021 Poprad	33.76 ± 0.013	7.60 × 10^5^	25.31 ± 0.002	1.81 × 10^6^	ND	ND	22.96 ± 0.004	1.30 × 10^8^

^*^ND not detected

### Comparison of wastewater levels of SARS-CoV-2 with numbers of COVID-19 cases

**Fig. 2:**
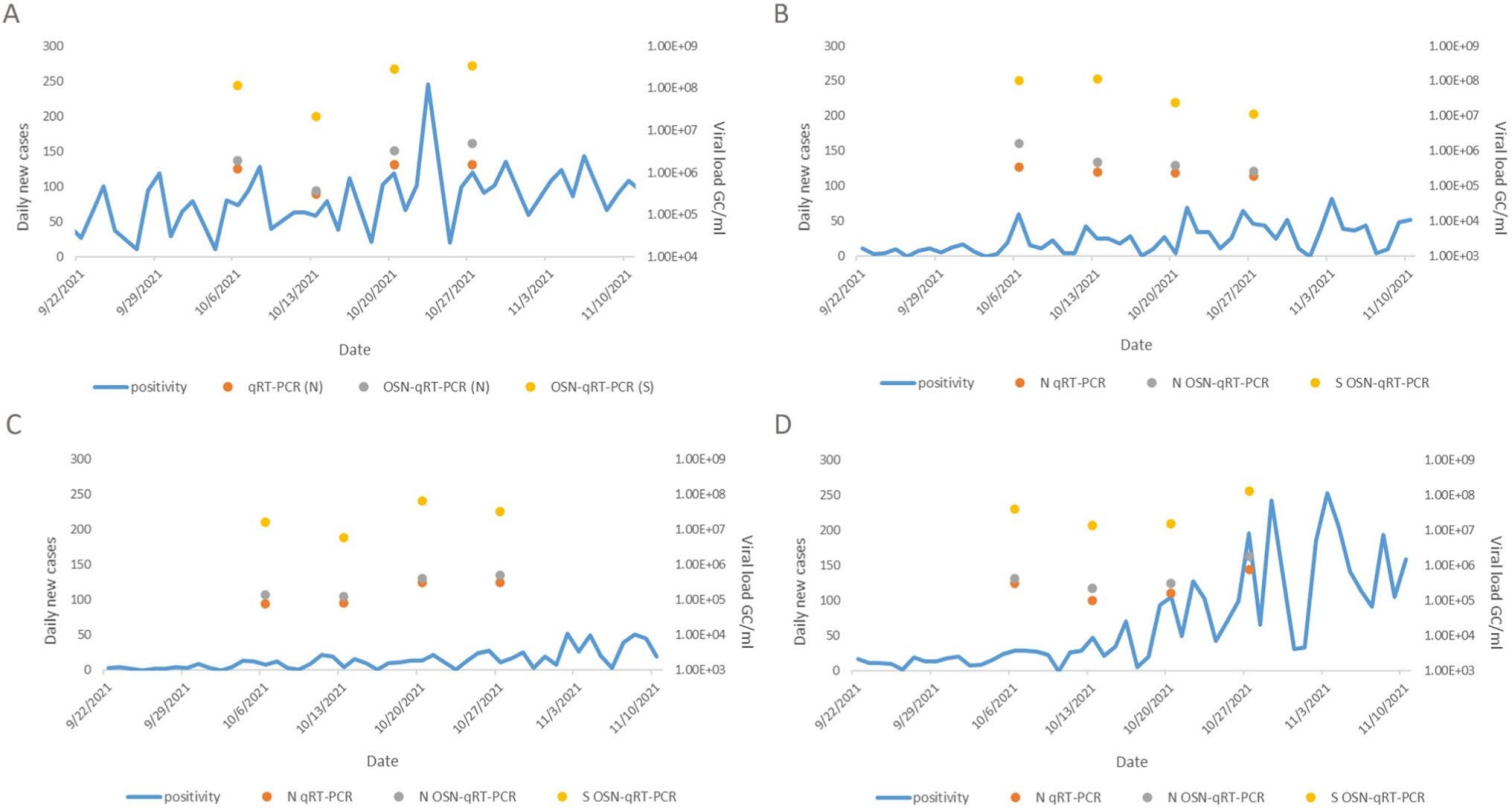
SARS-CoV-2 concentrations in wastewater compared with new clinical cases. Viral RNA concentrations in wastewater samples from October 6 to October 27, 2021, detected by qRT-PCR for N gene (orange dots), OSN-qRT-PCR for N gene (gray dots), and OSN-qRT-PCR for S gene (yellow dots) and new clinical cases from 22 September 2021 to 10 November 2021 in (**A**) Bardejov, (**B**) Kysucké Nové Mesto, (**C**) Komárno, and (**D**) Poprad counties (blue line)

Wastewater provides a sample of the infected population, including asymptomatic and pre-symptomatic individuals, also symptomatic but without clinical confirmation, and individuals who may have the disease but do not seek healthcare. In our study, SARS-CoV-2 concentrations in wastewater, detected by N qRT-PCR, N OSN-qRT-PCR, and S OSN-qRT-PCR, were compared with numbers of new clinical cases of COVID-19 from 22 September 2021 to 10 November 2021 in Bardejov, Poprad, Kysucké Nové Mesto, and Komárno counties. Results are presented in Fig. 2. A 3-, 4-, or 5-day temporal offset was applied. For Bardejov County, Pearson’s *r* was 0.71, 0.95, and 0.86 for N qRT-PCR, N OSN-qRT-PCR, and S OSN-qRT-PCR, respectively, with a 4-day temporal offset. For Poprad, Pearson’s *r* was 0.99 for all PCR assays with a 3-day temporal offset. For Komárno, Pearson’s *r* was 0.99, 0.99, and 0.80 for N qRT-PCR, N OSN-qRT-PCR, and S OSN-qRT-PCR, respectively, with a 5-day temporal offset. For Kysucké Nové Mesto, very low to moderate Pearson’s correlation between all PCR assays and 1 to 10 temporal offsets (0 < *r* ≤ 0.59) were detected.

## Discussion

One of the main aims of our study was the development of OSN-qRT-PCR assays based on N and S genes of SARS-CoV-2 and their mutual comparison as well as their comparison to already available ordinary qRT-PCR assays targeting the same fragments of *N* and *S* genes. The goal was to improve the detection sensitivity of SARS-CoV-2 in wastewater.

In order to test the developed assays in a correct way, the construction of reliable positive standards was necessary. We opted for the creation of two encapsidated RNA mimics based on PPV and obtained standard materials containing a fragment of *N* gene (ENRM) or *S* gene (ESRM) of SARS-CoV-2. The plant transient expression system applied for the preparation of these encapsidated mimics demonstrated to be low cost and sufficiently high yielding that a laboratory-scale production could offer enough positive standard material for many optimization experiments. Our results confirmed the findings of the previous studies (Chan et al. 2021; Peyret et al. 2022) which described the encapsidated mimics as stable particles that can be used as positive controls for detection assays. Such molecular standards are very important to validate accuracy and sensitivity of PCR assays utilized in wastewater screening.

For the concentration of SARS-CoV-2 from wastewater, various physical and chemical techniques are used. The PEG precipitation approach is able to concentrate the viruses by precipitating virus particles in the presence of NaCl. Although the exact mechanism is not certain, it is supposed that virus precipitation occurs similarly to PEG precipitation of proteins, in that water molecules are drawn from solution to hydrate PEG molecules, increasing the effective protein concentration, leading to insolubility and precipitation of proteins after reaching saturation (Ingham 1990; Yamamoto et al. 1970; LaTurner et al. 2021). According to our previous study (Rusková et al. 2022), we effectively used PEG precipitation approach coupled to NucleoSpin RNA Virus Kit for extraction of SARS-CoV-2 RNA from wastewater.

We developed OSN-qRT-PCR assays and demonstrated that they are fast, reliable, and sensitive. We made use of Luna universal probe one-step qRT-PCR kit, which according to Sapula et al. 2021 led to a higher detection efficiency for SARS-CoV-2 than TaqPath 1-Step multiplex master mix (no ROX). A one-step kit has the advantage of avoiding several time-consuming steps during the pipetting of the PCR master mix and, consequently, reducing potential cross-contamination. In fact, in a single tube, RNA is converted to cDNA by a reverse transcriptase and then a DNA-dependent DNA polymerase amplifies cDNA, allowing quantitation via qPCR. In a recent study, comparison between one-step qRT-PCR and two-step qRT-PCR approaches was evaluated, resulting in an improvement of sensitivity for the detection of SARS-CoV-2 in wastewater when the former type of approach was applied (Qiu et al. 2022).

The nested PCR alone has also the potential to increase detection sensitivity (Wang et al. 2020; Yip et al. 2020). This approach is based on two consecutive PCR amplifications where the product of the first PCR serves as a template in the second PCR. However, a great practical disadvantage of the conventional nested PCR is the risk of laboratory contamination by short amplified DNA fragments, which may take place at opening of the tubes and the transfer of the product of the first PCR to the second PCR. To overcome this, the development of a single-tube nested quantitative reverse transcription PCR is a valuable choice in order to have a sensitive and consistent PCR detection method. In addition, by the TaqMan probe, the whole process can be monitored continuously, in closed tubes, without the need of any subsequent, discontinuous analysis how it was the case in previous nested PCR assays applied to SARS-CoV-2 wastewater analysis (Rosa et al. 2020; Haramoto et al. 2020).

Wastewater surveillance should detect SARS-CoV-2 in a population before it is widespread. In fact, it was demonstrated that virus levels in wastewater are related to the numbers of local COVID-19 clinical cases (Wu et al. 2022). In our study, the virus levels in wastewater samples had usually a high or very high correlation with the clinical cases in the monitored area. The lowest correlation between viral levels in wastewater and clinical cases was recorded in Kysucké Nové Mesto County. This could be due to people’s mobility, as many local residents work in other regions and data of clinical cases are recorded in relation to the permanent residence of people and not to the place of testing. Moreover, variations in the amount of viral RNA excreted per person are also unknown (Lundy et al. 2021). Virus detection in wastewater has been reported at low incidence of reported human infections and mild, subclinical, or asymptomatic cases. Infected individuals may shed viruses into local sewage systems and contribute to virus circulation while remaining substantially undetectable by clinical surveillance (Ahmed et al. 2020).

According to Wu et al. 2022, trends of virus levels in wastewater precede by 4–10 days the numbers of clinical cases. In Poprad, Bardejov, and Komárno, we detected correlation with a 3-, 4-, or 5-day temporal offset, respectively. The difference between wastewater and clinical data could be due to underdiagnoses of asymptomatic or mildly symptomatic cases, limitations in clinical testing capacity, or a time delay between viral shedding and the onset of respiratory and other symptoms (Wu et al. 2022). These factors could vary slightly between the cities involved in our study. 

Hillary et al. 2020 collected data from various areas with the aim to critically assess the recent efforts on using wastewater surveillance to provide information relevant to public health; with a focus on SARS-CoV-2 surveillance, we can compare the results and conclude that we were able to achieve higher detection rates of SARS-CoV-2 in untreated wastewater due to the use of qRT-PCR and OSN-qRT-PCR assays. Wastewater-based epidemiology may find application in the future as an early warning system for virus outbreaks and monitoring the areas with viral outbreaks.

It was shown that the virus can be detected by qRT-PCR in wastewater several days before the number of clinical cases rises (Lundy et al. 2021; Wu et al. 2022). Based on our results, we can assume that we can detect the virus using OSN-qRT-PCR even earlier than qRT-PCR. Practically, OSN-qRT-PCR is more sensitive than qRT-PCR even more than digital PCR (Wang et al. 2020). The advancements with respect to previous studies (Fumian et al. 2010; Rusková et al. 2022; Sangsanont et al. 2022) regarded mainly the development of a new OSN-qRT-PCR oriented to N gene and the comparison of two OSN-qRT-PCR detection assays (based on N and S marker) using real samples from four wastewater treatments plants.

## Conclusion

Monitoring of urban wastewaters makes it possible to predict the extent of various diseases at a community level. To improve the detection of SARS-CoV-2 in wastewater, we developed new highly sensitive assays based on OSN-qRT-PCR. These assays, coupled to appropriate sample preparation, demonstrated high sensitivity, good laboratory convenience, and applicability to SARS-CoV-2 detection in wastewater. Due to their improved sensitivity, the developed OSN-qRT-PCR assays that may be useful in particular for analysis of wastewater samples with low virus concentrations in order to have a robust early warning system.

## Data Availability

All data generated or analyzed during this study are included in this published article.
